# Hydrogen Embrittlement Behavior of Laser Melting Deposited Ti-6Al-4V Alloy with Different Build Orientations

**DOI:** 10.3390/ma19091869

**Published:** 2026-05-01

**Authors:** Kejun Jiang, Junjun Jin, Yuxiang Cai, Zhihui Li, Sunmin Zou, Zhongyin Zhu, Guoqing Gou, Xiaohong Sun, Zhiyi Zhang

**Affiliations:** 1 Key Laboratory of Advanced Technologies of Materials, Ministry of Education, School of Materials Science and Engineering, Southwest Jiaotong University, Chengdu 610031, China; 2 School of Engineering Training Center, Southwest Jiaotong University, Chengdu 610031, China; 3 School of Materials Science and Engineering, Shanghai Jiao Tong University, Shanghai 180240, China; 4 CRRC Qingdao Sifang Co., Ltd., Qingdao 266111, China

**Keywords:** Ti-6Al-4V titanium alloy, laser melting deposition (LMD), hydrogen embrittlement, build orientation

## Abstract

This study investigates the hydrogen embrittlement susceptibility of laser melting deposition (LMD)-produced Ti-6Al-4V alloy with different build orientations (0°, 45°, 90°) through electrochemical hydrogen charging, slow strain rate testing, and microstructural characterization. Ti-6Al-4V alloys are widely used in marine and offshore engineering, where cathodic protection and corrosion reactions can generate hydrogen, leading to hydrogen ingress and potential embrittlement. Results show that prolonged hydrogen charging induces hydride formation, α-phase fragmentation, and β-phase dissolution, significantly degrading corrosion resistance and mechanical properties. Hydrogen embrittlement susceptibility exhibits notable anisotropy: elongation reductions for 0°, 45°, and 90° specimens are 40.1%, 40.8%, and 29.4%, respectively. The relatively superior resistance observed in the 90° orientation may be associated with its single-layer structure and more uniform dimple distribution. In contrast, the multilayer interfaces in other orientations are likely to serve as preferential sites for hydrogen accumulation, which may contribute to the increased embrittlement susceptibility. This research reveals the failure mechanism of LMD Ti-6Al-4V in hydrogen environments and supports its application in marine engineering.

## 1. Introduction

Titanium alloys are characterized by their low density, high strength, excellent corrosion resistance, high fracture toughness, and low coefficients of thermal expansion and elastic modulus, making them widely used in aerospace, petrochemical, biomedical, and other fields [1,2]. They are also increasingly employed in deep-sea applications, such as propellers, heat exchangers, condensers, and pressure hulls. In particular, the use of titanium alloys in deep-sea pressure hulls represents an emerging field of development. Compared to conventional steel materials, titanium alloys exhibit superior resistance to seawater corrosion, rendering them more suitable for marine environments. Ti-6Al-4V titanium alloy, the first practical titanium alloy developed in the United States in 1954, has become the flagship alloy in the titanium industry due to its well-balanced comprehensive properties, formability, and weldability. It accounts for more than 50% of the total usage of titanium alloys [3].

The high melting point and low thermal conductivity of Ti-6Al-4V titanium alloy classify it as a refractory metal, presenting significant challenges for conventional metallurgical methods in processing large and complex structures, thereby limiting its broader industrial application. The emergence of additive manufacturing technology has provided an effective pathway to address this challenge. In essence, this technology is an advanced manufacturing method based on the principle of discrete stacking, enabling rapid, mold-free forming of three-dimensional solid components through the sequential layer-by-layer accumulation of material. Among the various metal additive manufacturing processes, laser metal deposition, electron beam deposition, and selective laser melting (SLM) are considered representative [4,5]. Specifically, the electron beam deposition process must be conducted in a high-vacuum environment, and its inherent energy characteristics along with relatively large melt pool dimensions generally result in components with inferior accuracy and surface quality. While SLM technology excels in producing high-precision components with complex internal structures, its build chamber size is typically limited, and the resulting parts often exhibit significant residual stresses, usually requiring subsequent heat treatment for relief; additionally, the utilization rate of powder material is relatively low. Laser melting deposition (LMD), a coaxial powder-feeding technology, offers high manufacturing efficiency and the capability to produce large, complex components, making it one of the most promising metal additive manufacturing techniques. This advanced process constructs intricate three-dimensional metal parts through layer-by-layer material deposition, effectively overcoming the difficulties associated with Ti-6Al-4V titanium alloy processing. The technology enables the integrated manufacturing of complex parts for various application fields of titanium alloy. It not only produces components with favorable performance but also reduces production lead time, cost, and material waste. This provides a new technical pathway for low-cost, short-cycle, and near-net-shape manufacturing of large and complex titanium alloy structures, showing significant application potential in the production of deep-sea equipment such as pressure hulls and robotic arm supports. The high cooling rate during the LMD process refines the microstructure of titanium alloy and generates high-density dislocations, resulting in a lamellar structure and dislocation substructures that enhance the hydrogen trap capacity within the material. This contributes to superior hydrogen embrittlement resistance compared to alloys produced by conventional methods [6,7,8]. With further advancements in titanium alloy processing technology, laser melting deposition is expected to see expanded application in manufacturing these metals and their alloys, particularly for forming complex structural components [9,10,11].

Environmentally assisted degradation (EAD) induced by hydrogen environments, particularly hydrogen embrittlement (HE), significantly impacts the physico-mechanical properties of structural materials. Hydrogen can penetrate into materials through various pathways during manufacturing or service, leading to substantial performance deterioration. When titanium alloy components operate in extreme environments rich in halides or hydrogen, the combined action of applied stress and corrosive products or diffusible hydrogen atoms promotes the formation of brittle hydrides. This results in loss of mechanical properties and brittle fracture [12,13,14,15,16], ultimately causing catastrophic premature failure. According to incomplete statistics, more than 20% of incidents involving titanium equipment are attributed to hydrogen-induced damage and failure. Recent studies [17,18,19,20,21] have shown that hydrogen embrittlement susceptibility is highly dependent on the initial microstructural characteristics of the material. Ti-6Al-4V produced by conventional processes exhibits a dual-phase microstructure consisting of α (HCP) and β (BCC) titanium phases [22,23]. In contrast, Ti-6Al-4V alloys fabricated by laser melting deposition (LMD) typically consist of fine α-phase grains with a small amount of β-phase. This difference in microstructure may lead to distinct hydrogen embrittlement susceptibility in LMD-produced materials compared to those manufactured by conventional methods [24,25].

A.-M. Alvarez et al. investigated the effect of hydrogen on the mechanical behavior of a metastable bcc β-titanium alloy (Ti-3Al-8V-6Cr-4Zr-4Mo) [26]. The results indicated that hydrogen embrittles the β-phase titanium alloy. Crack formation occurs through a mechanism involving stress-induced hydride formation and cleavage, whereby these cleaved hydrides act as nucleation sites for cracks that propagate into the β-phase. In (α + β) titanium alloys, the presence of the β-phase makes the dual-phase microstructure more susceptible to hydrogen embrittlement compared to single-phase α microstructures. Liu et al. [27] studied the influence of high hydrostatic pressure on the stress corrosion cracking (SCC) of Ti-6Al-4V alloy in a 3.5% NaCl solution at low temperature. The results showed that under both 0.1 MPa and 20 MPa pressures, the alloy exhibited mixed fracture characteristics with fine shallow dimples, cleavage facets, and quasi-cleavage surfaces. At 20 MPa, the high hydrostatic pressure exacerbated the SCC of Ti-6Al-4V by promoting titanium dissolution and reducing the resistance of the oxide film. Furthermore, hydrostatic pressure facilitated the formation of δ-hydrides at the α/β interface in the Ti-6Al-4V alloy. Kudiiarov et al. investigated the effects of hydrogen on additively manufactured Ti-6Al-4V alloy from perspectives such as hydrogen adsorption and desorption behavior [28,29], microstructural evolution [30], as well as defect structures and mechanical properties [31,32]. Gong et al. [33] studied the corrosion behavior of electron-beam-melted (EBM) Ti–6Al–4V specimens with building orientation angles of 0°, 45°, 55°, and 90° relative to the deposition direction. The results indicated that the corrosion resistance of the EBM alloy in 1M HCl solution improved slightly in the order of 45°, 90°, 55°, and 0°. This variation was attributed to grain boundary density and β-phase composition, which change with building direction. Yuan et al. [34] experimentally elucidated the influence of building direction on the microstructure and macroscopic response of selective laser melting (SLM)-produced Ti-6Al-4V alloy under compressive loading at different strain rates. The results demonstrated that the building direction had little effect on the fracture morphology of specimens under quasi-static compression but significantly influenced the fracture patterns under dynamic loading. It is noteworthy that, in addition to process parameters, heat treatment, and loading conditions, the layer-by-layer deposition characteristic of the LMD process also leads to anisotropy in the mechanical properties of the material, thereby affecting its hydrogen embrittlement susceptibility. However, research on the mechanism of hydrogen interaction and the influence of building orientation on laser-melting-deposited (LMD) Ti-6Al-4V titanium alloy remains relatively limited. Thus, further investigation is warranted.

This study focuses on the hydrogen embrittlement (HE) susceptibility of Ti-6Al-4V alloy fabricated by laser melting deposition (LMD) with different build orientations. Through gradient dynamic electrochemical hydrogen charging experiments combined with microstructural characterization and electrochemical testing, the diffusion behavior of hydrogen in LMD Ti-6Al-4V and its underlying mechanisms were thoroughly investigated. Meanwhile, slow strain rate tests (SSRT) were conducted to compare the differences in HE susceptibility of additively manufactured Ti-6Al-4V specimens with different build orientations (0°, 45°, and 90° relative to the deposition direction) under in situ hydrogen charging. The structure–property relationship between hydrogen embrittlement behavior and mechanical performance was elucidated from the perspective of microstructural evolution. Compared with existing studies, this work systematically reveals the coupling effect of build orientation and hydrogen-induced damage in LMD Ti-6Al-4V and provides an interpretation of the anisotropic hydrogen embrittlement behavior by correlating hydrogen diffusion characteristics with microstructural features and mechanical response. In particular, the possible role of layer interfaces as hydrogen trapping sites and their potential influence on crack initiation and propagation are discussed, offering additional insight into the failure mechanism of additively manufactured titanium alloys in hydrogen-containing environments.

## 2. Material and Methods

### 2.1. Sample

**Figure 1:**
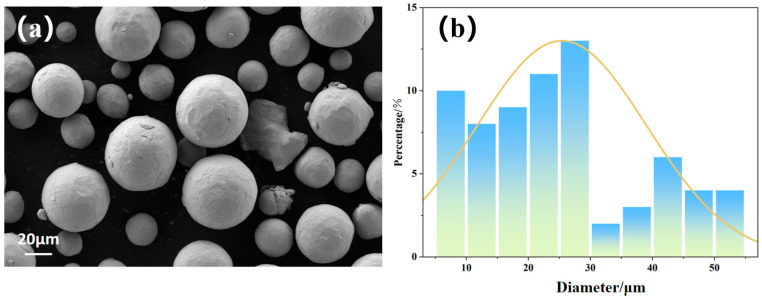
(**a**) Ti-6Al-4V powder particle morphology; (**b**) particle size statistics.

**Figure 2:**
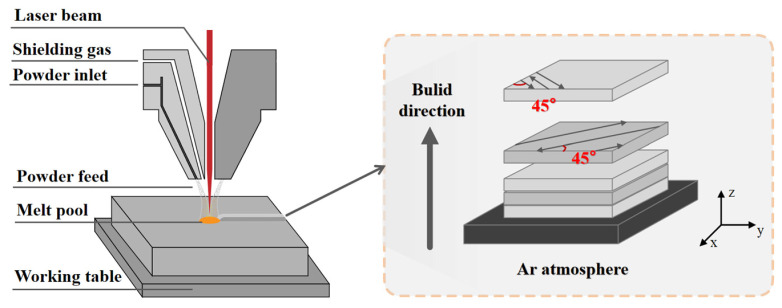
Schematic diagram of laser metal deposition process.

The raw material used in this study is Ti-6Al-4V alloy powder for LMD processing, and its chemical composition is listed in Table 1. The powder morphology and particle size distribution are shown in Figure 1a,b. The particle size statistics were obtained from 10 randomly selected SEM images, each covering an area of approximately 220 × 300 µm^2^ and containing about 40–60 particles, yielding a total of over 500 particles analyzed. Figure 2 shows the schematic diagram of the laser metal deposition (LMD) process. Under the protection of high purity (99.99%) Ar gas, the laser power, scanning speed, scanning speed (contour), and powder feeding rate are optimized to 3840 W, 1000 mm/min, 800 mm/min, and 26 g/min, respectively. To improve manufacturing efficiency, a high laser power of 5 kW was employed. During the deposition process, contour scanning was first performed along the boundary, followed by infill scanning at an angle of 45° relative to the boundary. In addition, the scanning direction was rotated by 90° layer by layer, maintaining a 45° angle with respect to the boundary frame. A Ti-6Al-4V block with dimensions of 50 mm × 200 mm × 200 mm was first fabricated by LMD, and specimens with dimensions of 10 mm × 10 mm × 10 mm were then cut from the deposited block for subsequent experiments.

**Figure 3:**
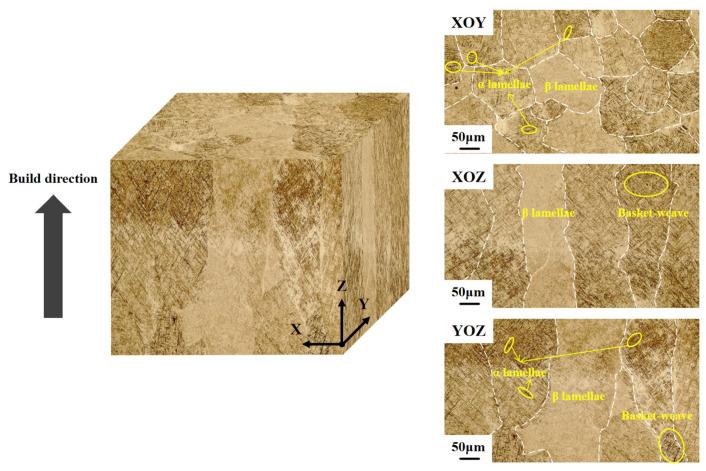
Three-dimensional metallographic diagram of Ti-6Al-4V titanium alloy prepared by LMD.

The three-dimensional metallographic structure of Ti-6Al-4V was observed using an optical microscope (AxioCam MRc5, Carl Zeiss AG, Oberkochen, Germany), as shown in Figure 3. The top of the sample is mainly composed of equiaxed primary β grains, and the side shows a microstructure dominated by columnar β grains. However, whether columnar or equiaxed grains are observed, the internal microstructure consists of a bimodal structure and basketweave structure. Electrochemical pre-hydrogen charging was conducted using an aqueous solution of 1 mol/L H_2_SO_4_ with 2 g/L CH_4_N_2_S as the electrolyte, at a current density of 100 mA/cm^2^. Here, CH_4_N_2_S (thiourea) was employed as a poisoning agent to inhibit the recombination of hydrogen atoms into hydrogen molecules, thereby promoting hydrogen ingress into the titanium alloy. During the experiment, hydrogen was introduced through a single-side charging method. After charging, the samples were promptly rinsed with distilled water and ethanol and then stored in a desiccator for subsequent testing. To investigate the evolution of hydrogen-induced damage in LMD-produced Ti-6Al-4V titanium alloy, a gradient of hydrogen charging durations (12, 24, and 48 h) was applied to simulate varying degrees of hydrogen degradation.

After hydrogen charging, several groups of samples were microscopically characterized. The surface corrosion was removed by grinding and polishing the hydrogen charging surface. The samples were etched using Keller reagent (1% HF + 1.5% HCl + 2.5% HNO_3_ + 95% H_2_O, volume fraction) for 10–20 s. The metallographic micrographs of Ti-6Al-4V samples after hydrogen charging were observed using an optical microscope (AxioCam MRc5, Carl Zeiss AG, Oberkochen, Germany). Then, the phase composition of titanium alloy samples before and after hydrogen charging was analyzed by an X-ray diffractometer (Empyrean, PANalytical, Almelo, The Netherlands). The XRD test was conducted using a Cu target, with a diffraction angle (2θ) range of 5–90° and a scanning speed of 2°/min.

### 2.2. Electrochemical Test

The electrochemical tests were conducted using a CS310 M potentiostat (Wuhan Corrtest Instruments Corp., Ltd., Wuhan, China). A conventional three-electrode system was employed. The working electrode was a titanium alloy sample after different durations of hydrogen charging, with an exposed area of 1 cm^2^. A platinum plate was used as the counter electrode, and a saturated calomel electrode (SCE) served as the reference electrode. The electrolyte was 3.5% NaCl. Firstly, the sample was immersed in the solution for half an hour to ensure the stability of the system, and then the open circuit potential was measured. After the open circuit potential reached a stable state, electrochemical impedance spectroscopy (EIS) and polarization curve tests were carried out. The EIS measurements were performed over a frequency range of 10^5^ Hz to 10^−2^ Hz with an AC amplitude of 10 mV. The polarization curves were recorded at a scanning rate of 0.002 V/s within a potential range of −1.5 V to +1.5 V. All electrochemical tests were conducted in accordance with ASTM G5 and ASTM G106 standards.

### 2.3. Slow Strain Rate Tensile Test

**Figure 4:**
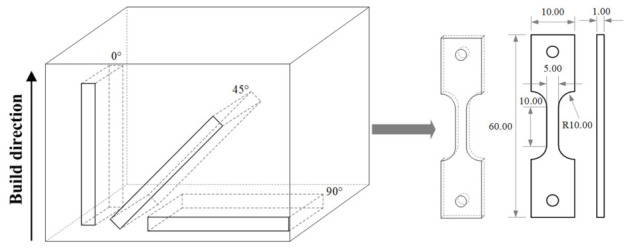
Orientation diagram and sample size diagram (unit: mm).

**Figure 5:**
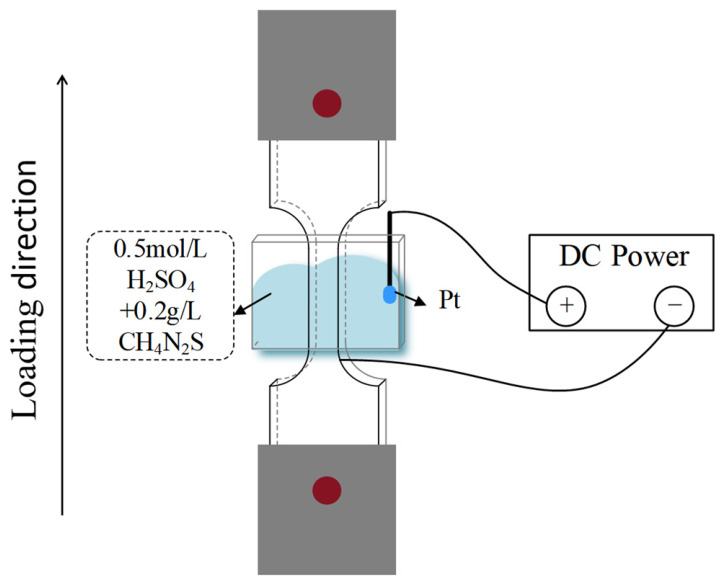
Schematic diagram of the slow strain rate test under in situ hydrogen charging.

For each build orientation—parallel (0°), 45° angle, and perpendicular (90°) to the stacking direction (Figure 4)—six specimens were extracted. In each orientation, three specimens were tested in air, serving as the control group, while the other three were subjected to in situ electrolytic hydrogen charging during slow strain rate tensile testing. All mechanical property data reported in this study are presented as the average values of the three parallel specimens. The tests were carried out at a slow strain rate of 1 × 10^−5^ s^−1^ in accordance with ISO 7539-7 (Corrosion of metals and alloys—Stress corrosion testing—Part 7: Slow strain rate testing). The hydrogen charging environment consisted of 0.5 mol/L H_2_SO_4_ + 0.2 g/L CH_4_N_2_S. This experimental design was adopted to investigate the hydrogen embrittlement sensitivity of the laser melting deposition (LMD)-produced Ti-6Al-4V alloy under different build orientations. All tests were completed at room temperature (see Figure 5). After the tensile test, the samples were ultrasonically cleaned with deionized water and ethanol in turn and dried with high-purity nitrogen. The fracture morphology was systematically characterized by field emission scanning electron microscopy (FE-SEM, Sigma 500, Carl Zeiss AG, Germany).

## 3. Result and Discussion

### 3.1. Microstructure Analysis

**Figure 6:**
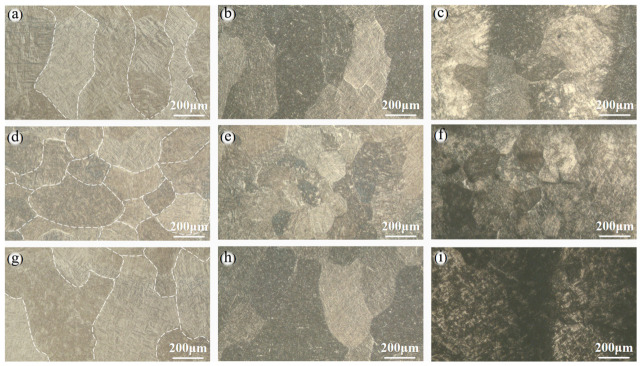
The metallographic diagram of Ti-6Al-4V after hydrogen charging in different construction directions: (**a**) 0° sample hydrogen charging 12 h, (**b**) 0° sample hydrogen charging 24 h, (**c**) 0° sample hydrogen charging 48 h, (**d**) 45° sample hydrogen charging 12 h, (**e**) 45° sample hydrogen charging 24 h, (**f**) 45° sample hydrogen charging 48 h, (**g**) 90° sample hydrogen charging 12 h, (**h**) 90° sample hydrogen charging 24 h, (**i**) 90° sample hydrogen charging 48 h.

Figure 6 illustrates the microstructural evolution of the Ti-6Al-4V titanium alloy fabricated by laser-directed energy deposition (LMD) after different hydrogen charging durations (12 h, 24 h, 48 h). After 12 h of hydrogen charging, the material retains a typical α + β dual-phase basket-weave morphology [35], with only slight local contrast variations observed, indicating that hydrogen atoms remain predominantly in solid solution and have not yet induced significant phase transformation or hydride precipitation.

When the hydrogen charging time is extended to 24 h, the microstructure exhibits a pronounced darkening phenomenon. The introduction of hydrogen leads to the fragmentation of α laths and refinement of the microstructure. The interfaces between α phases and α/β phases serve as effective trapping sites for hydrogen, while the β phase acts as a short-path channel for hydrogen transport [30]. This microstructural refinement and the accumulation of hydrogen at interfaces can contribute to the observed reduction in ductility at 24 h, as trapped hydrogen weakens interfacial cohesion and promotes microvoid nucleation during deformation. However, the morphology of α laths and the characteristics of α/β interfaces remain relatively intact, with no evident secondary phase precipitates observed at grain boundaries or phase boundaries. This indicates that although the hydrogen content has increased at this stage, hydrogen still exists in the form of solid solution or metastable clustering, and large-scale hydride precipitation has not yet occurred. Consequently, the loss of mechanical properties at 24 h, while clearly measurable, remains less severe than that at longer charging times.

As the hydrogen charging time further increases to 48 h, a noticeable microstructural transformation takes place. Particularly in the 45°-direction specimen, distinct black precipitated phases are clearly observed at grain boundaries and phase boundaries, indicating that significant hydride precipitation has occurred at this stage. In contrast, while the 0°- and 90°-direction specimens also exhibit a certain degree of darkening and localized precipitation features, the overall extent is less pronounced than that observed in the 45° direction. This microstructural difference correlates well with the lower embrittlement susceptibility measured in the 0° and 90° orientations, suggesting that the degree of hydride precipitation at interfaces is a key factor governing the anisotropic hydrogen embrittlement behavior. Thus, with increasing hydrogen charging time, hydrogen gradually transitions from a solid-solution state to interfacial enrichment, ultimately inducing hydride precipitation in a manner that exhibits a certain build direction dependence. This progressive microstructural evolution—from hydrogen trapping to hydride precipitation—underpins the monotonic degradation of mechanical properties and the anisotropic embrittlement susceptibility observed in this study.

**Figure 7:**
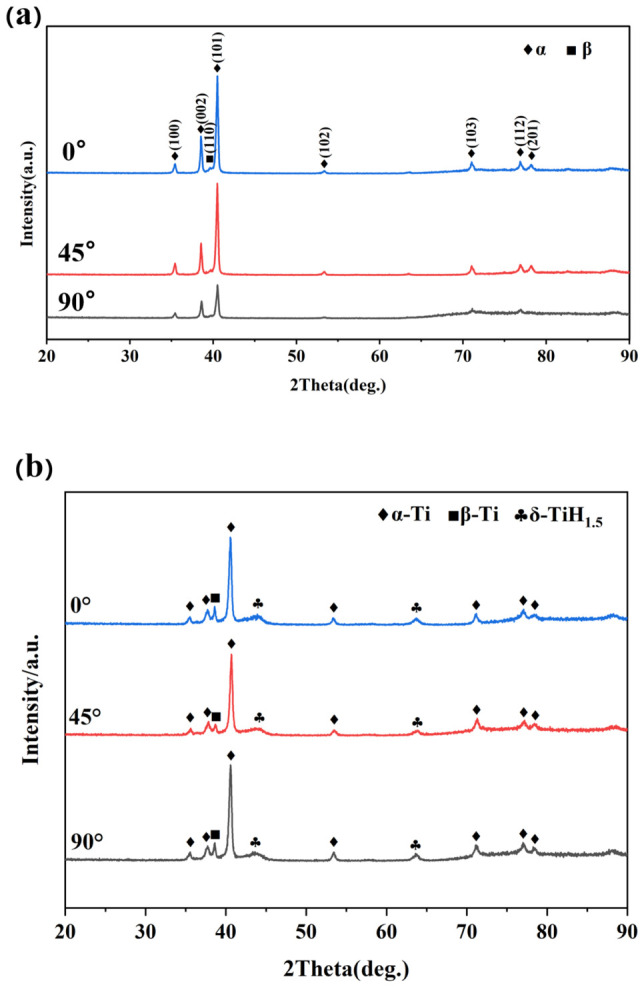
XRD patterns of Ti-6Al-4V samples with different construction directions: (**a**) original state, (**b**) 48 h after hydrogen charging.

Figure 7a shows the XRD patterns of the three build orientations (0°, 45°, 90°). It can be observed that the diffraction peak positions of the three orientations are generally consistent, all predominantly featuring the α-Ti phase. Weak characteristic peaks of the β phase can be observed in the range of 38–40°, and no significant new diffraction peaks appear, indicating that the phase composition of the material remains consistent across different build orientations. In view of the fact that a large number of hydride precipitation characteristics have been observed in the microstructure after 48 h of hydrogen charging, in order to further verify from the phase point of view, samples with different construction directions were selected for XRD testing and analysis under this hydrogen charging condition. As shown in Figure 7b, the diffraction peak position and relative strength of Ti-6Al-4V samples prepared by LMD technology in three directions are basically the same, indicating that different deposition directions have no significant effect on the phase composition after hydrogen charging. However, new diffraction peaks appear obviously in the XRD spectra of the samples after 48 h hydrogen charging treatment. Studies have shown that [36,37,38,39] different hydrogen contents will induce the formation of three different titanium hydride phases: when the hydrogen content is low, a metastable γ phase with a face-centered tetragonal structure is formed; under the condition of medium hydrogen content, the δ phase with face-centered cubic structure is formed; when the hydrogen content is high, the metastable ε-hydride with face-centered tetragonal structure is formed. Compared with the uncharged sample, the diffraction peak shape of the sample changed significantly after hydrogen charging for 48 h. The α-Ti diffraction peak near 37.5° and the β-Ti diffraction peak near 38.5° were transformed into two adjacent small peaks in the range of 37~38° after hydrogen charging. This change is attributed to the hydrogen atoms entering the lattice gap between the α phase and the β phase, causing the lattice volume expansion and the increase of the interplanar spacing, resulting in a low angle shift of the diffraction peak, accompanied by the peak shape splitting caused by lattice distortion [40,41,42]. In addition, after hydrogen charging, a wide and low diffraction peak appeared near 43°, and a new small peak appeared near 64°. Compared with the standard PDF card, these two diffraction peaks correspond to the characteristic diffraction positions of the (200) crystal plane and the (220) crystal plane of the δ-TiH2 hydride, respectively. The appearance of the above new peaks indicates that after 48 h hydrogen charging treatment, there is obvious hydride precipitation in the sample. Combined with the change of peak intensity, it can be inferred that the formation of hydrides continues to consume the α-Ti matrix, which is consistent with the rule of hydride precipitation after hydrogen charging of titanium alloy reported in the literature [43,44,45].

### 3.2. Electrochemical Analysis

**Figure 8:**
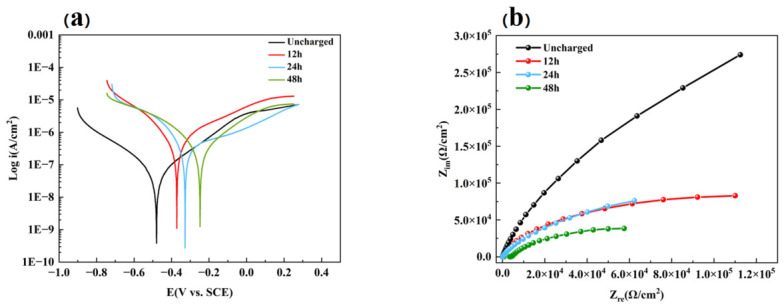
(**a**) Polarization curve and (**b**) Nyquist plot of Ti-6Al-4V under different hydrogen charging times.

Figure 8 shows the potentiodynamic polarization curves (a) and Nyquist plots (b) of Ti-6Al-4V alloy in 3.5 wt% NaCl solution after different hydrogen charging durations. From the electrochemical curves, parameters such as the corrosion potential (E_corr_) and corrosion current density (I_corr_) of TC4 titanium alloy after different hydrogen charging times were obtained by fitting (Table 2). The fits show that E_corr_ shifts positively from −480.3 mV (uncharged) to −248.5 mV (48 h), while I_corr_ varies non-monotonically, initially rising, then decreasing at 24 h, and increasing again after 48 h. All samples generally exhibit passivation behavior, which is characteristic of titanium alloys in neutral environments due to the formation of a stable oxide film. However, compared with the uncharged and short-time hydrogen-charged samples, the specimen with prolonged hydrogen charging shows a more pronounced passivation region. Some studies have shown that under certain conditions [46,47], a hydride layer can provide a protective effect to some extent, similar to that of a natural passive film. When hydrogen extensively enriches on the surface of a titanium alloy and forms hydrides, a dense hydride film can develop on the surface. Some researchers believe that this layer can, to a certain degree, replace or enhance the protective function of the passive film, thereby inhibiting corrosion [48]. The progressive ennoblement of E_corr_ observed here is consistent with such a surface modification effect.

This distinct passivation behavior can be attributed to the combined effects of hydrogen-induced microstructural evolution and surface film modification. On the one hand, hydrogen ingress promotes the formation of hydride phases and increases lattice distortion, which may facilitate the rapid formation of a compact passive film during anodic polarization. On the other hand, the presence of hydrogen can alter the defect structure and electronic properties of the oxide film, enhancing its short-term stability and thus leading to an apparent passivation feature in the polarization curve. Meanwhile, the extension of hydrogen charging time leads to a gradual shift of the cathodic polarization curve toward higher current density, indicating enhanced cathodic reaction kinetics. This change is likely related to the increased hydrogen concentration near the surface, which reduces the energy barrier for cathodic reactions and promotes charge transfer processes. The Nyquist diagram of the titanium alloy in neutral chloride-containing solution shows an incomplete single capacitive arc, which is a typical electrochemical impedance response of titanium alloys under such conditions [49,50,51]. A magnified view of the high-impedance region reveals a depressed semicircle, corresponding to the charge transfer process at the electrode/electrolyte interface. The radius of the capacitive arc reflects the charge transfer resistance of the material, and a larger radius indicates better corrosion resistance [52]. With increasing hydrogen charging time, the radius of the capacitive arc decreases monotonically. In particular, after 48 h of hydrogen charging, the impedance is significantly reduced, indicating that hydrogen accelerates the corrosion process and deteriorates the corrosion resistance of the material.

### 3.3. Slow Strain Rate Test

**Figure 9:**
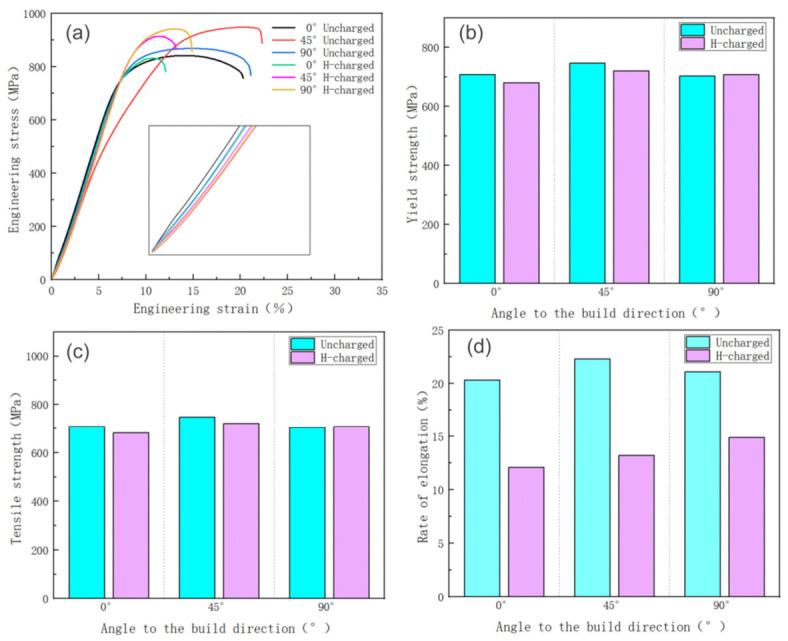
Slow strain rate tensile results of Ti-6Al-4V titanium alloy under different conditions: (**a**) engineering stress–strain curve; (**b**) yield strength; (**c**) tensile strength; (**d**) elongation.

Figure 9 presents the slow strain rate tensile results of Ti-6Al-4V samples with different orientations under unhydrogenated (in air) and hydrogenated conditions. It can be seen from the engineering strain–stress curve in Figure 6a that the slope of the stress–strain curve of each sample in the elastic deformation stage is not much different, indicating that the entry of hydrogen atoms has no obvious effect on the elastic deformation behavior of titanium alloy. For yield strength and tensile strength, the strength of the 0° sample and 45° sample under the hydrogen charging condition is slightly lower than that in air. The strength of the 90° sample increased slightly under the condition of simultaneous hydrogen charging. Comparing the strength of samples with different orientations in the same environment, it can be seen that the strength and elongation of 45° samples are better than those of 0° and 90° samples. This is because during the laser metal deposition process, the rapid cooling of the molten pool promotes the epitaxial growth of the β crystal along the thermal gradient direction, forming a strongly textured columnar crystal, which subsequently transforms into α′ martensite and retains the same orientation characteristics. For the 0° specimen, the tensile load is parallel to the columnar crystal growth direction, and the grain boundary and the α/β phase boundary become the rapid slip channel of dislocations, resulting in early plastic deformation. In the 90° specimen, the load is perpendicular to the columnar grain boundary, and the crack is easy to propagate along the phase boundary. In contrast, the load direction of the 45° sample has a certain angle with the columnar crystal, and the dislocation needs to cross more grain boundaries and α laths, which significantly improves the deformation resistance. At the same time, the limitation of cross slip delays the necking, so the comprehensive mechanical properties are better.

In this study, the hydrogen embrittlement sensitivity ($I_{HE}$) was defined by the elongation after fracture of titanium alloy specimens, as shown in the following equation [53,54]:

(1) $$I_{HE}=\frac{\delta_{0}-\delta_{H}}{\delta_{0}}\times 100\%$$

In the formula, $\delta_{0}$ is the elongation of the uncharged sample, and $\delta_{H}$ is the elongation of the material after hydrogen charging. The hydrogen embrittlement sensitivity c is positively correlated with the value of $I_{HE}$, that is, the larger the value of $I_{HE}$, the higher the hydrogen embrittlement sensitivity, and the smaller the value of $I_{HE}$, the smaller the hydrogen embrittlement sensitivity. It can be seen from Figure 9d that the $I_{HE}$ 90° sample along the Z-axis direction is 29.4%, while the 0° and 45° samples are 40.4% and 40.8%, respectively.

The results show that hydrogen has an effect on the hydrogen embrittlement sensitivity of Ti-6Al-4V alloy manufactured by LMD at different angles, while the SCC sensitivity in the 0° and 45° directions is more significantly affected by hydrogen. Typical hydride phases such as δ-TiH_x_ themselves possess extremely low fracture toughness. According to reference [55], their room-temperature fracture toughness is only 2.2 MPa·m^1/2^. In contrast, the fracture toughness of non-hydrided pure α-Ti is approximately 60 MPa·m^1/2^ [56]. The difference exceeds an order of magnitude. These “soft but brittle” hydride phases readily initiate microcracks under applied loads, particularly at interfaces such as grain boundaries and phase boundaries, making them preferred sites for crack initiation [57,58]. In the materials prepared by laser melting deposition (LMD), the microstructure characteristics are often significantly different due to different sampling directions. This structural anisotropy is closely related to the forming process. Comparing the samples with different orientations, it can be found that the structure of the 90°-oriented sample is composed of a single cladding layer, while the 0° and 45° samples involve multi-layer stacking. Studies have shown that the number of cladding layers has a significant effect on the macroscopic mechanical behavior of the material. In addition, the rapid melting characteristics during laser processing will introduce a significant residual stress field. This kind of stress concentration effect may increase the local stress intensity factor, which in turn aggravates the sensitivity of stress corrosion cracking (SCC), and especially promotes the nucleation and propagation of cracks at the interface between layers. Experimental observations show that LMD components oriented along the stacking direction are more prone to brittle fracture under a hydrogen-induced damage environment, which eventually leads to service failure.

### 3.4. Secondary Crack Analysis

**Figure 10:**
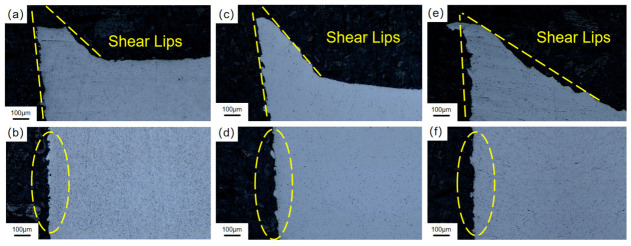
Macroscopic fracture of Ti-6Al-4V after SSRT under air condition by LMD: (**a**,**b**) 0° specimen; (**c**,**d**) 45° specimen; (**e**,**f**) 90° specimen.

The transverse macroscopic fracture morphology of Ti-6Al-4V titanium alloy with different orientations formed by LMD in an air environment is shown in Figure 10. There is an inclined section at the edge of the fracture of all oriented specimens, which is about 45° ± 5° with the principal stress axis, that is, the shear lip, which is derived from the plastic slip deformation on the maximum shear stress plane before fracture. No secondary crack initiation was observed on the side of the specimen. These phenomena can further confirm that the fracture mode of Ti-6Al-4V in the air environment is ductile fracture.

**Figure 11:**
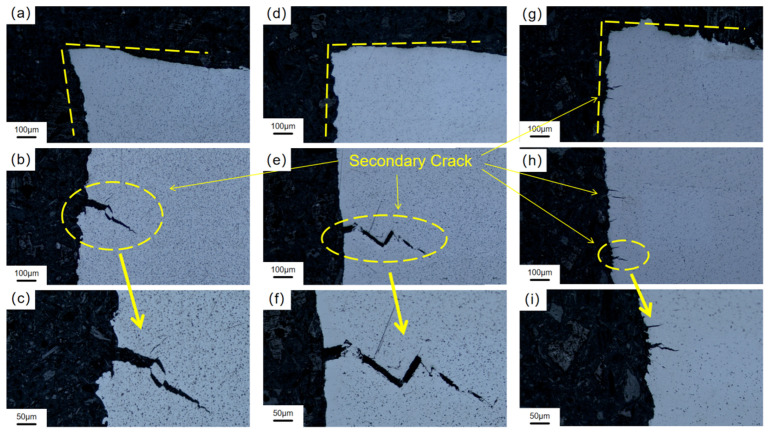
Macroscopic fracture of Ti-6Al-4V after SSRT under hydrogen charging condition manufactured by LMD: (**a**–**c**) 0° specimen; (**d**–**f**) 45° specimen; (**g**–**i**) 90° specimen.

As shown in Figure 11, compared with the air environment, the fracture edges of the three oriented specimens under hydrogen charging conditions show significant flattening characteristics, and secondary cracks perpendicular to the tensile stress direction appear on the side. The typical ‘Z’-shaped crystallographic propagation path can be observed through the local amplification of cracks (Figure 11c,f,i), indicating that dynamic hydrogen charging induces hydrogen-induced brittle transgranular fracture of Ti-6Al-4V titanium alloy. It is worth noting that the secondary crack size of the 90°-orientation sample is significantly smaller than that of other orientations (Figure 11c,f), which confirms that it has relatively excellent plastic retention ability in the hydrogen-filled environment. This conclusion, together with the smaller hydrogen embrittlement sensitivity index (29.4%) of the 90°-orientation sample mentioned above, verifies the influence of the number of stacking layers on hydrogen embrittlement sensitivity [59].

### 3.5. Fracture Morphology Analysis

**Figure 12:**
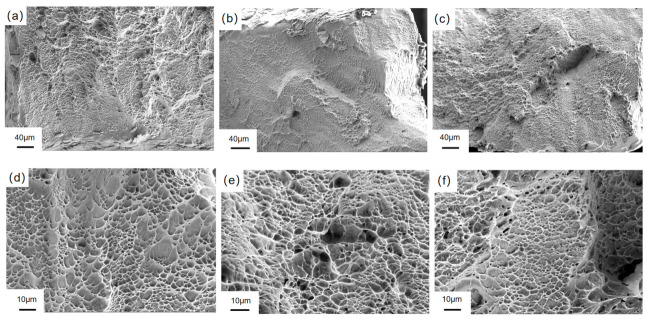
The fracture morphology of Ti-6Al-4V after SSRT fabricated by LMD in air: (**a**) 0° specimen; (**b**) 45° specimen; (**c**) 90° specimen; (**d**) 0° specimen; (**e**) 45° specimen; (**f**) 90° specimen.

**Figure 13:**
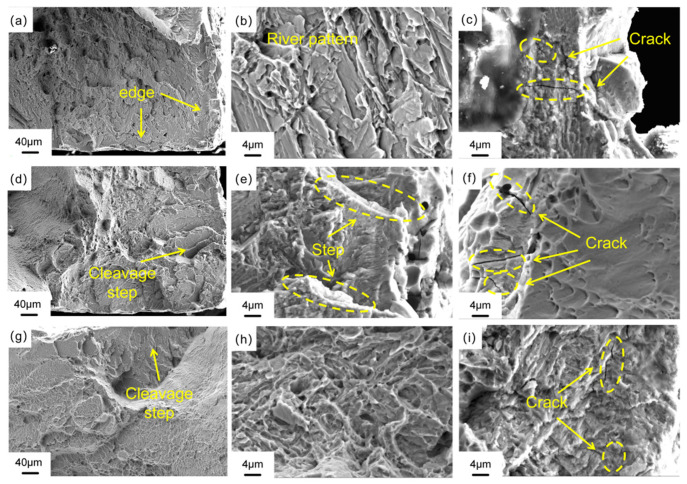
The fracture morphology of Ti-6Al-4V after SSRT fabricated by LMD under the condition of hydrogen charging: (**a**–**c**) 0° specimen; (**d**–**f**) 45° specimen; (**g**–**i**) 90° specimen.

After ultrasonic cleaning and vacuum drying of the fracture of the specimen after slow strain rate tensile (SSRT) testing, the fracture morphology was systematically characterized by scanning electron microscopy (SEM) at magnifications of 500×, 2000×, and 5000×, respectively. As shown in Figure 12, the fracture morphology analysis of Ti-6Al-4V alloy prepared by LMD in an air environment shows that compared with the hydrogen charging conditions, the oriented samples (0°, 45°, 90°) show a more flat fracture surface. The high-magnification SEM observation shows that the fracture presents a uniformly distributed equiaxed dimple structure, and the dimple size and distribution characteristics of the samples with different orientations are basically the same. This phenomenon confirms that in the air environment, the fracture mechanism of the three oriented samples is typical transgranular ductile fracture. Figure 13 reveals the difference in fracture behavior of samples with different orientations under hydrogen charging conditions. The low-magnification SEM observation (Figure 12a,d,g) showed that all the oriented samples (0°, 45°, 90°) showed a mixed fracture mode of edge cleavage fracture and central dimple coexistence. The specific performance is as follows: the fracture edge region presents typical cleavage steps and tearing edges, indicating that hydrogen-induced embrittlement is significant; a certain amount of dimples are distributed in the central region, which confirms that the region still maintains a certain plastic deformation ability. The spatial distribution characteristics of this fracture morphology can be attributed to the gradient effect of hydrogen diffusion: the edge region is directly exposed to the hydrogen charging solution, and the hydrogen concentration reaches a critical value, causing hydrogen-induced cleavage fracture; due to the limitation of hydrogen diffusion time, the hydrogen concentration in the central region is lower than the critical value, thus maintaining the ductile fracture mechanism. Relatively speaking, the dimples in the central region of the 90°-oriented sample are relatively small, dense, and evenly distributed; this microstructure characteristic is consistent with the macroscopic mechanical properties test results, that is, the elongation at break (14.9%) of the 90° sample in the hydrogen-filled environment is higher than that of the 0° (12.1%) and 45° (13.2%) samples, indicating that it has better resistance to hydrogen embrittlement. High-magnification microscopic analysis further reveals the microscopic mechanism of hydrogen-induced damage: all oriented specimens exhibit typical brittle fracture characteristics caused by hydrogen embrittlement, including cleavage steps, river patterns, and transgranular cracks. Through the comparative analysis of SEM images, it is found that the crack of the 45°-oriented sample is the most significant, and the crack morphology of the 0°-oriented sample is slightly smaller than that of the 45°-oriented sample, but it still has obvious visibility. The crack size of the 90° specimen is significantly smaller than that of the other two orientation specimens at the same magnification. This phenomenon is also consistent with the previous calculation results (0°: 40.4%, 45°: 40.8%, 90°: 29.4%), which further confirms that the 90°-orientation sample exhibits the best hydrogen embrittlement resistance in three orientations.

A limitation of this study is that hydrogen concentration was not directly measured; instead, charging time was used as the experimental variable. The actual hydrogen content in LMD Ti-6Al-4V may be influenced by microstructural anisotropy and phase distributions. Consequently, the observed orientation-dependent embrittlement susceptibility might reflect not only differences in bulk hydrogen uptake but also local hydrogen redistribution effects. Future work employing direct hydrogen mapping techniques is needed to clarify these contributions.

## 4. Conclusions

**Figure 14:**
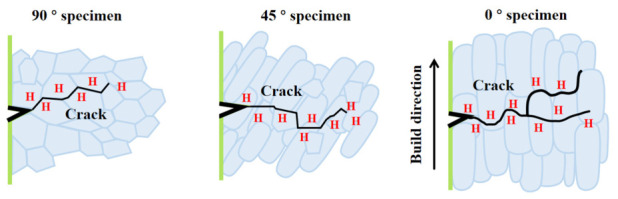
Schematic of the mechanism for crack growth in Ti-6Al-4V with different building orientations under hydrogen charging.

This study systematically investigated the formation behavior of hydrides, electrochemical corrosion characteristics, anisotropic mechanical response, and hydrogen embrittlement sensitivity of LMD-fabricated Ti-6Al-4V alloy. The effects of hydrogen charging time and stacking direction on microstructure evolution and performance degradation were analyzed through electrochemical and mechanical evaluations. The results indicate that hydrogen charging significantly affects both the microstructural features and performance of the alloy, leading to enhanced corrosion activity and degradation of mechanical properties with increasing charging time. The following conclusions can be drawn:

1. Hydrogen initially dissolves in the α-Ti matrix, inducing lattice defects, followed by the precipitation of acicular and continuous hydrides at the α/β interfaces. The presence of hydrogen reduces the phase transformation energy barrier, thereby promoting the α → βH transition. Concurrently, these hydrides, acting as cathodic phases, disrupt the protective surface passive film and establish galvanic cells. With prolonged hydrogen charging time, the cathode activity of the Ti-6Al-4V alloy is enhanced while its impedance is reduced, collectively leading to the acceleration of corrosion.
2. The mechanical properties of Ti-6Al-4V alloy fabricated by LMD exhibit anisotropy. The 45° specimen demonstrates the best overall performance, as dislocations must traverse multiple cladding layers and grain boundaries during deformation. Although the 90° specimen exhibits the lowest plasticity, its single-track cladding structure facilitates the formation of uniformly fine dimples, resulting in superior resistance to crack propagation. This phenomenon is attributed to the synergistic effect between the cladding layer architecture and the orientation of columnar grains.
3. As shown in Figure 14, under hydrogen charging conditions, the material transitions to a mixed fracture mode due to hydrogen-induced bond weakening, internal pressure, and “Z”-shaped microcracks, with all orientations exhibiting hydrogen embrittlement. The 0° and 45° specimens, where multi-layer interfaces accumulate both hydrogen and stress, demonstrate the highest hydrogen embrittlement susceptibility (>40%) and are at greater risk of stress corrosion cracking (SCC). In contrast, the 90° specimen exhibits lower susceptibility (29.4%) and retains more ductility, as its single-track structure effectively suppresses hydrogen diffusion.

## Figures and Tables

**Table 1:** Chemical composition of Ti-6Al-4V powder used for LMD (mass fraction %).

Al	V	Fe	N	O	C	Ti
5.5–6.7	3.6–4.5	<0.30	<0.05	<0.20	<0.10	Bal

**Table 2:** The corrosion potential and corrosion current density of TC4 specimens after different hydrogen charging durations.

Hydrogen Charging Time (h)	0	12	24	48
E_corr_ (mV)	−480.3	−371.8	−328.0	−248.5
I_corr_ (A/cm^2^)	8.43 × 10^−8^	5.75 × 10^−7^	1.67 × 10^−7^	4.31 × 10^−7^

## Data Availability

The original contributions presented in this study are included in the article. Further inquiries can be directed to the corresponding authors.
